# Stakeholder-Oriented Firms Have Feelings and Moral Standing Too

**DOI:** 10.3389/fpsyg.2022.814624

**Published:** 2022-02-21

**Authors:** Katinka J. P. Quintelier

**Affiliations:** Department of Management and Organization, School of Business and Economics, Vrije Universiteit Amsterdam, Amsterdam, Netherlands

**Keywords:** moral standing, experience attributions, agency attributions, stakeholder theory, stakeholder orientation, profit orientation

## Abstract

A central claim in stakeholder theory is that, if we see stakeholders as human beings, we will attribute higher moral standing or show more moral consideration to stakeholders. But would the same hold for firms? In this paper, I apply the concepts of humanization and moral standing to firms, and I predict that (1) individuals attribute higher moral standing to stakeholder-oriented than to profit-oriented firms, because (2) individuals attribute more experience (such as feelings) to stakeholder-oriented than to profit-oriented firms. Five experiments support these predictions across different operationalizations of stakeholder and profit orientations. The analyses show that moral standing attributions are not fully explained by attributions of agency (such as thinking) to firms, or by attributions of experience or agency to human stakeholders (instead of firms). By unearthing the importance of experience attributions for moral standing attributions to firms, this work provides novel insights in ongoing legal, philosophical and public debates related to firms’ moral standing. The findings also bring the debate about firms’ moral standing to the heart of stakeholder theory, and lead to new normative and descriptive research questions about the interests of firms and their stakeholders.

## Introduction

Certain court decisions have given corporations (for-profits as well as non-profits) moral standing, or rights and protections that are typically given to human beings only. In 2012, a representative of AT&T argued in court that AT&T had privacy rights because AT&T—as an organization rather than as a collection of individuals—could be embarrassed (FCC v. AT and T Inc., 2011). At times, such arguments resonate: the U.S. Supreme Court has granted corporations legal rights and protections, by virtue of these corporations’ capacity for having feelings (Garrett, 2014; Iuliano, 2015). At other times, court officials deny that corporations have moral standing. For instance, a Philippine court argued that corporations are “not entitled to moral damages” because they have “no feelings, no emotions and no sense” (LBC v. CA, 1994).

How can we explain this apparent disagreement? The above examples suggest that, when individuals perceive corporations as having a mind—human-like mental characteristics such as feelings, embarrassment, emotions or sense –, they are likely to attribute moral standing to these corporations. But this raises other questions. Which corporations are more likely to elicit mind attributions? And which dimensions of mind, this is, what kind of attributed human-like mental characteristics, are likely to elicit moral standing attributions? To investigate this, I apply the psychology of humanization, and how it underlies moral standing attributions, to one specific set of corporations: firms, or for-profits.

In this paper I empirically investigate the effect of a business orientation—a set of firm-level prescriptions about how a firm (a for-profit corporation) should balance stakeholders’ interests and profit (cf. Jones et al., 2007; Phillips, 2011; Freeman et al., 2020; Quintelier et al., 2021)—on mind and moral standing attributions to the firm. I compare stakeholder- and profit-oriented firms, as they are of growing importance for current theory (Freeman, 1984; Freeman et al., 2010, 2020; Parmar et al., 2010) and practice (Harrison et al., 2020). I then distinguish between two dimensions of mind—experience and agency—because empirical work has shown that experience attributions, compared to agency attributions, are stronger predictors of lay people’s moral standing attributions (Gray et al., 2012). Individuals attribute more experience to an entity if they perceive the entity to be more able to have emotions, feelings, or consciousness; and individuals attribute more agency to an entity if they perceive the entity to be more able to have intentions, free will, or a mind of its own (Gray et al., 2012). Insights from the psychology of humanization lead to the first prediction that individuals attribute more experience to a firm when they perceive the firm as stakeholder-oriented than when they perceive the firm as profit-oriented. This leads to the second prediction that higher experience attributions to a firm increase moral standing attributions to the firm. Five experiments support these predictions, across four different operationalizations of a stakeholder and profit orientation. Eliminating alternative explanations (Bernerth and Aguinis, 2016), the findings also show that moral standing attributions to a firm are not fully explained by attributions of agency to the firm. Moral standing attributions to a firm are also not fully explained by experience or agency attributions to the human stakeholders of the firm.

This work contributes to ongoing debates in business ethics about corporations’ moral standing. Both in the US and Europe, courts have decided that corporations have rights and protections similar to the rights and protections human beings have (Friedman, 2020). In contrast, according to Silver (2019), business ethicists tend to reject the idea that corporations have moral standing, and they tend focus on discussion about corporate moral responsibility instead. In addition, some court decisions granting moral standing to corporations have led to outrage among the general public (Levitt, 2015; Blair and Pollman, 2017). Despite this ongoing legal, public and professional debate, there is scant research about the psychological processes influencing moral standing attributions to corporations (for one exception, see Mentovich et al., 2016). This study is the first to empirically support the role of experience attributions in explaining individuals’ moral standing attributions to corporations; as such, it unearths experience attributions as a relatively overlooked argument in the debate.

The findings also open up novel research questions in stakeholder theory. The question of corporate moral standing turns out to be particularly important for stakeholder-oriented firms. If firms increasingly present themselves as more stakeholder-oriented (Harrison et al., 2020), this might gradually shift public opinion or court decisions on firms’ moral standing. This raises questions about how individuals will reason about the rights of the firm vs. the interests of its stakeholders (Quintelier et al., 2021). In sum, future work in business ethics and stakeholder theory can benefit from integrating additional insights from the psychology of humanization.

## Theory and Hypotheses

When introducing the concept of moral standing (French, 1979; Donaldson, 1982; Werhane, 1985; Velasquez, 2003; Blair, 2015; Sepinwall, 2015; Hess, 2018), it is important to distinguish moral standing from responsibility. Moral psychologists speak of moral standing as the extent to which an entity can be harmed or wronged, and is deserving of moral consideration, for instance in the form of rights and protection (cf. Sytsma and Machery, 2012; Piazza et al., 2014; Silver, 2019). In contrast, responsibility means that the entity is held responsible for its actions (cf. Ashforth et al., 2020), that it has obligations or duties, or that it can be blamed for its actions (Manning, 1984; Velasquez, 2003). As noted by Hess (2013), moral standing and responsibility are sometimes conceptually conflated, but they are not the same. The difference can perhaps be made intuitively clear by pointing out that we show consideration for babies—they have moral standing—but we do not hold them responsible for their actions. Work in business ethics covers firms’ responsibility in great depth (Velasquez, 2003; Ruiz-Palomino et al., 2011; Hess, 2013, 2014, 2016), but questions about firms’ moral standing currently receive less attention in business ethics (for one exception, see Silver, 2019).

To investigate the psychological processes underlying moral standing attributions to corporations, it makes sense to look at firm-level constructs such as a business orientation. Legal scholars distinguish between for-profits and non-profits, and have suggested that non-profits have more rights than for-profits (Garrett, 2014). Management scholars also identify different firm-level constructs. Relevant to the current argument are stakeholder-oriented firms, which balance the interests of a broad group of stakeholders, and profit-oriented firms, which see (short-term) profit maximization as the goal of business (Jones et al., 2007; Phillips, 2011; Parmar et al., 2019; Freeman et al., 2020). The relevance of firm-level constructs in legal discourse begs the question if individuals also attribute more moral standing to stakeholder-oriented than to profit-oriented firms. Stakeholder orientations are also increasingly relevant, as in 2019 181 CEO’s of leading U.S. American firms committed to leading their firms for the benefit of all stakeholders (Harrison et al., 2020). In addition, stakeholder theory is increasingly adopted as a theoretical lens across disciplines and countries (Laplume et al., 2008; Parmar et al., 2010; Fares et al., 2021). Because of this practical and theoretical relevance, I investigate the effect of a stakeholder vs. profit orientation on moral standing attributions to a firm. The next section explains the concepts of a stakeholder and profit orientation.

### Stakeholder and Profit Orientation

I conceptualize the concept of a business orientation, which consists of a stakeholder and a profit orientation, in line with thinking in stakeholder theory (Jones et al., 2007; Freeman et al., 2010, 2020). A business orientation describes the extent to which a firm balances its stakeholders’ interests or prioritizes (short-term) financial performance, such as profit (Jones et al., 2007; Phillips, 2011; Freeman et al., 2020). Stakeholder interests and financial performance can go together (Jones et al., 2018), which we can denote as synergy (Tantalo and Priem, 2016). In this case, firms can be stakeholder-oriented and focus on balancing stakeholders’ interests, assuming that this strategy leads to long-term profit (Ruiz-Palomino et al., 2011; Jones et al., 2018); or firms can be profit-oriented and focus on maximizing profit, assuming that this strategy requires balancing stakeholders’ interests (Jensen, 2002). In addition, firms may also be faced with trade-offs, especially in the short term (Harrison and Bosse, 2013). In this case, firms can score high on stakeholder elements and low on profit elements, or high on profit elements and low on stakeholder elements (Brickson, 2005). In the absence of trade-offs or synergies, firms can also focus on stakeholder (profit) elements without being explicit about profit (stakeholder) elements. The experiments in this paper describe a firm’s orientation highlighting synergy (experiment 5), highlighting trade-offs (experiments 1 and 3), and without explicit trade-offs or synergy (experiments 2, 4, and 5). In all experiments, when a firm focuses on stakeholder elements, it is called stakeholder-oriented, and when a firm focuses on profit elements, it is called profit-oriented.

Stakeholder-oriented firms consider a broad range of stakeholders’ interests (Freeman et al., 2010). This leads to stakeholder interactions that are motivated by moral concerns (Donaldson, 1999) such as fairness concerns (Phillips, 2003; Bosse and Phillips, 2016). Examples of such interactions are more informal, trust-based contracts, solving problems through collaboration (Bridoux and Stoelhorst, 2014), and long-term cooperative relationships with stakeholders (Bridoux and Stoelhorst, 2014; Jones et al., 2018). In contrast, profit-oriented firms pursue the short-term maximization of their financial performance, such as profit (Friedman, 2007). Profit is argued to be more relevant for shareholders than for non-shareholder stakeholders (Greenley and Foxall, 1997, p. 277), but financial performance is also important for firms that do not have shareholders. A profit purpose leads to instrumental interactions with stakeholders, such as more formal and short-term contracts, hard bargaining, replacing stakeholders, and using legal procedures to solve problems (Brickson, 2005; Jones et al., 2007; Bridoux and Stoelhorst, 2014).

### The Effect of a Business Orientation on Firms’ Moral Standing

Stakeholder scholars argue, and find, that there is a positive relationship between a stakeholder orientation, and seeing *stakeholders* as human beings who have mind and moral standing (Donaldson and Preston, 1995; McVea and Freeman, 2005; Harris and Freeman, 2008; Newkirk and Freeman, 2008; Purnell and Freeman, 2012; Quintelier et al., 2021). For instance, stakeholder scholars develop a stakeholder theory of the firm, which describes business as an activity consisting of human beings, whose interests are morally legitimate (Freeman, 1994; McVea and Freeman, 2005; Newkirk and Freeman, 2008; Freeman et al., 2010). However, building on the psychology of humanization, I argue that there is also a positive relationship between a stakeholder orientation and seeing the *firm* as a human-like being, with a human-like mind, and having moral standing in the form of rights and protection. More specifically, I argue that a business orientation influences experience attributions to the firm which, in turn, influence moral standing attributions to the firm.

Psychologists speak of mind attributions when individuals attribute human-like mental characteristics to an entity (Epley et al., 2007; Waytz et al., 2010; Gray et al., 2012). Previous work in organizational theory and organizational psychology has focused on mind attributions to organizations (Ashforth et al., 2020), or to their stakeholders (Quintelier et al., 2021). However, mind consists of two dimensions—experience and agency. Individuals attribute more experience to an entity if they perceive the entity to possess a greater ability for having emotions, feelings, or consciousness; and individuals attribute more agency to an entity if they perceive the entity to possess a greater ability to have intentions, free will, or a mind of its own (Gray et al., 2012). For instance, individuals tend to attribute experience but not agency to fetuses, and individuals tend to attribute agency but not experience to robots (Gray et al., 2007). The distinction between agency and experience is important: While previous work in business ethics (French, 1979; Donaldson, 1982; Blair, 2015) and in psychology (Rai and Diermeier, 2015) tends to focus on firm agency, empirical work has shown that experience attributions, compared to agency attributions, are stronger predictors of lay people’s moral standing attributions (Gray et al., 2012). This paper therefore focuses on experience attributions to firms, and their effects on moral standing attributions to firms.

To develop empirical predictions, I build on insights from the psychology of humanization. A first insight is that an entity’s interactions with other entities influence mind attributions to the entity (Epley et al., 2007; Waytz et al., 2010; Gray et al., 2012). In general, individuals attribute more experience to entities that, or who, interact in more prosocial ways with others than to entities that, or who, are less prosocial. For instance, participants who read about an outgroup (a group of people belonging to a different group than oneself) helping people in need attribute more experience (e.g., grief) to this outgroup than individuals who are not presented with information about the helping behavior of this outgroup (Delgado et al., 2012; Davies et al., 2018). Profit cues are also related to perceived prosociality. Specifically, a profit-motivated practice tends to be seen as less helpful to society than the same practice in the absence of profit motives (Bhattacharjee et al., 2017). As a consequence, for-profit practices of an entity may decrease individuals’ experience attributions to that entity.

The above arguments can be applied to firms. Stakeholder-oriented firms tend to interact with their stakeholders in a prosocial manner, while profit-oriented firms tend to incentivize their stakeholders with financial cues (Bridoux and Stoelhorst, 2014). Descriptions of the goals and practices of stakeholder-oriented firms, compared to profit-oriented firms, are therefore likely to increase experience attributions to the organizations. This is in line with empirical findings by Rai and Diermeier (2015, 2019) that experience attributions to firms can vary; but these authors did not investigate which firm-level elements increased experience attributions. The above arguments lead to the more specific hypothesis that individuals attribute more experience to a firm they perceive as stakeholder-oriented than to a firm they perceive as profit-oriented.

*H1: Individuals attribute more experience to a firm when they perceive the firm as stakeholder-oriented than when they perceive the firm as profit-oriented.*

A second relevant insight from the psychology of humanization is that experience attributions are strongly related to moral cognition (Gray et al., 2012). In the field of moral cognition, the theory of dyadic morality suggests that moral cognition consists of a dyadic template (Gray et al., 2012). This means that individuals’ moral cognition is triggered if they perceive an interaction between two entities who exhibit the capacity for experience and agency. For instance, participants (exhibiting experience and agency themselves) are less willing to harm another entity with more experience than an entity with less experience (Gray and Wegner, 2009).

In particular, experience attributions have been found to positively and consistently relate to moral standing (Gray et al., 2007). For instance, when experimentally increasing participants’ experience attributions to monkeys or aliens, these participants also attribute more moral standing to these monkeys or aliens (Sytsma and Machery, 2012). The effect of experience attributions on moral standing attributions is more consistent than the effect of agency attributions. As an example, Sytsma and Machery (2012) do find that agency attributions influence moral standing, but not in situations where individuals strongly empathize with an entity. Likewise, when comparing individuals’ experience and agency attributions to living and dead people, animals and God, moral standing correlates more with experience attributions than with agency attributions (Gray et al., 2007; Piazza et al., 2014). These findings support that higher experience attributions to an entity lead to higher moral standing attributions to the entity.

How would this play out for firms? Rai and Diermeier (2015, 2019) find that attributions of experience to firms compared to entrepreneurs lead to higher sympathy. Sympathy positively relates to moral consideration (Hoffman, 2008; Decety and Cowell, 2015; Yoder and Decety, 2018). This suggests that the relation between experience attributions and moral standing also extrapolates to firms. These arguments lead to the following prediction:

*H2: When individuals attribute more experience to a firm, they will attribute more moral standing to that firm.*

Combining the first and second hypothesis leads to the third hypothesis:

*H3: Individuals attribute more moral standing to a firm they perceive as stakeholder-oriented than to a firm they perceive as profit-oriented. This is mediated by individuals’ experience attributions to the firm.*

## Overview of Experiments

The aim is to test theory about the causal relationships between variables. Vignette experiments are well-suited for this, because experiments allow to manipulate the independent variable and test causal relationships, while vignettes add realism to the description (Aguinis and Bradley, 2014). The vignettes described an organization by systematically combining stakeholder or profit elements. The experimental paradigms for experiments 1–4 were used in a previous study (Quintelier et al., 2021). Experiment 5 was designed to increase ecological validity and to expand the effects to a different operationalization of a business orientation.

Experiments 1 and 2 tested the effect of orientation on experience attributions to firms. In experiment 1, the vignettes featured trade-offs between stakeholder and profit elements while in experiment 2 the vignettes did not feature trade-offs. Experiments 3 and 4 tested the effect of orientation on moral standing attributions to firms, mediated by experience attributions. The vignettes again featured trade-offs (exp. 3) or no trade-offs (exp. 4). Experiment 5 increased the ecological validity by making use of a mock website, and it expanded the findings to new operationalizations of a stakeholder and profit orientation featuring synergy.

## Experiments 1 and 2

### Sample and Design

Based on previous experiments (Rai and Diermeier, 2015; Quintelier et al., 2021), I expected a large effect size, which requires a sample size of 84 for ANOVA according to (conservative) calculations with the help of the power analysis program G*Power (Faul et al., 2007). Participants were randomly assigned to the stakeholder- or profit-oriented vignette, thus using a one-way between subjects design. For experiment 1, 97 participants, recruited in a course setting in a university in the Netherlands, completed the experiment (55 men, *M*_*age*_ = 26.39; *SD* = 16.28; 48 stakeholder-oriented; Supplementary Data Sheet 1). After removing participants who gave a wrong answer to an attention check, as described below, 82 participants remained (47 men, *M*_*age*_ = 25.09; *SD* = 5.71; 40 stakeholder-oriented). For experiment 2, participants were recruited on www.clickworker.com—a German website similar to Amazon’s Mechanical Turk (MTurk). Participants were compensated with 0.6€, which is in line with policies on clickworker. Ninety-three participants completed the experiment (47 men, *M*_*age*_ = 35.01; *SD* = 15.34; 47 stakeholder-oriented; Supplementary Data Sheet 2). After removing participants who gave a wrong answer to an attention check, 89 participants remained (45 men, *M*_*age*_ = 34.36; *SD* = 14.03; 44 stakeholder-oriented).

### Procedure and Measures

Participants were introduced to the experiment and asked to confirm that they had read the information and agreed to proceed. They were then asked to read the vignette description that started with “Alpha is a grocery retailer which recently built a new store in your neighborhood. Alpha sells products that you buy on a weekly basis.” This background information was chosen because being a potential customer of a grocery store is a realistic situation for most participants. Alpha was then either described as stakeholder-oriented or profit-oriented. In experiment 1, the *stakeholder-oriented* (profit-oriented) vignette read:

Alpha is committed to improving its *stakeholders’ welfare* (financial performance), because Alpha believes this is *the morally right thing to do* (necessary to be a successful business). This commitment to *stakeholder welfare and doing what is morally right* (financial performance and being a successful business) translates into practices that improve *stakeholder welfare* (financial performance), also if these practices result in lower *financial performance* (stakeholder welfare). Specifically, Alpha *invests in relationships with its suppliers, rather than switching to the supplier who asks the lowest price* (switches to the supplier who asks the lowest price rather than investing in relationships with its suppliers). In addition, Alpha constantly optimizes its *operations to increase customer satisfaction, also if this leads to lower profits* (profits, also if this leads to lower customer satisfaction). When new skills are needed, Alpha *trains its current employees, instead of replacing them with skilled applicants who ask the same wage* (replaces its employees with skilled applicants who ask the same wage, instead of training current employees). Finally, Alpha resolves conflicts with the local community *through collaboration rather than via legal procedures* (via legal procedures rather than through collaboration).

In experiment 2, the *stakeholder-oriented* (profit-oriented) vignette only explicitly stated stakeholder (profit) elements. For experiment 2, the *stakeholder-oriented* (profit-oriented) vignette read:

Alpha is committed to improving its *stakeholders’ welfare* (financial performance) because Alpha believes this is *the morally right thing to do* (necessary to be a successful business). This commitment to *stakeholder welfare and doing what is morally right* (financial performance and being a successful business) translates into practices that improve *stakeholder welfare* (financial performance). Specifically, Alpha *invests in relationships with its suppliers* (switches to the supplier who asks the lowest price). In addition, Alpha constantly optimizes its operations to increase *customer satisfaction* (profits). When new skills are needed, Alpha *trains its current employees* (replaces its employees with skilled applicants who ask the same wage). Finally, Alpha resolves conflicts with the local community *through collaboration* (via legal procedures).

The vignette description was immediately followed by two factual attention check questions, whereby participants had to select the statement that was part of the description they had just read. Kane and Barabas (2019) argue that factual attention checks (this is, questions about key elements of the experiment) enable researches to identify individual participants who are inattentive to the experiment, while these factual attention checks have little or no consequences for the treatment effects. For the first question, the participants could choose between: “Alpha is a grocery retailer selling products that you buy on a weekly basis” (correct); “Alpha has job openings consistent with your career goals,” “Alpha is a corporation that you might include in your investment portfolio.” For the second question, participants could choose between “Alpha is committed to improving its financial performance, because Alpha believes this is necessary to be a successful business” (correct for profit-oriented), “Alpha is committed to improving its stakeholders’ welfare, because Alpha believes this is necessary to be a successful business” and “Alpha is committed to improving its stakeholders’ welfare, because Alpha believes this is the morally right thing to do” (correct for stakeholder-oriented). Participants who gave a wrong answer to any of the attention checks were removed from the analysis.

In order to measure experience, I adapted the two experience items from Waytz et al. (2010) Participants answered (on a seven-point Likert scale from “*not at all”* to “*very much”*): to what extent does Alpha: “experience emotions” and “have consciousness.” The scale’s reliability varied but could not be improved by dropping items (exp. 1: α = 0.699; exp. 2: α = 0.829). To rule out alternative explanations that participants interpreted these items as aspects of agency, I also measured agency. I adapted the three agency items from Waytz et al. (2010). Participants were asked: to what extent does Alpha “have intentions”; “have free will”; “have a mind of its own.” The scale’s reliability again varied but could not be improved by dropping items (exp. 1: α = 0.652; exp. 2: α = 0.872). Because the reliability of the scales was low in experiment 1, I also conducted the analysis with each item separately.

The survey was followed by manipulation checks. To see if the vignettes manipulated the organization’s orientation as intended, participants were asked to indicate on a slider scale from 0 to 100 to what extent they thought the organization was long-term vs. short-term oriented, and to what extent the firm prioritized stakeholders vs. firm-level performance. These features (time orientation and priorities) are not literally in the vignette description, but are part of a firm’s orientation as described in the “Theory and Hypotheses” section. I also asked if the vignettes were realistic (“The description was realistic”) and imaginable (“I had no difficulty imagining this situation”), on a scale from 1 (strongly disagree) to 7 (strongly agree). This was followed by demographic variables (year of birth, gender, nationality).

### Results

A MANOVA on the manipulation checks revealed that the manipulation worked (see Table 1). Only in experiment 2, significantly more U.S. Americans were assigned to the profit-oriented vignette than to the stakeholder-oriented vignette; I therefore controlled for nationality in the analysis. There were no further differences in demographics or control questions between the stakeholder- and profit-oriented vignette, meaning that both vignettes were perceived as equally realistic and imaginable.

**TABLE 1:** Means, standard deviations, and significance levels of manipulation checks and control variables in experiments 1–5.

Experiment	Variable	Stakeholder	Profit	

		*M* (*SD*)	*M* (*SD*)	*F* (*p*)
1 (trade-off)	Long-term	70.62 (23.69)	25.67 (18.86)	*F*(1, 81) = 90.81 (< 0.001)
	Stakeholders	73.35 (20.41)	21.92 (20.0)	*F*(1, 81) = 132.44 (< 0.001)
2 (no trade-off)	Long-term	62.48 (13.47)	39. 60 (28.64)	*F*(1, 88) = 23.07 (< 0.001)
	Stakeholders	60.79 (19.80)	34.36 (29.78)	*F*(1, 88) = 24.21 (< 0.001)
	Nationality	19 United States.	29 United States	*F*(1, 88) = 4.15 (= 0.045)
3 (trade-off)	Long-term	75.32 (17.13)	46.34 (26.34)	*F*(1, 50) = 20.10 (< 0.001)
	Stakeholders	74.73 (18.59)	39.720 (25.81)	*F*(1, 50) = 28.98 (< 0.001)
	Nationality	13 German	8 German	*F*(1, 50) = 5.47 (= 0.023)
4 (no trade-off)	Long-term	57.36 (17.77)	43.55 (21.99)	*F*(1, 163) = 19.42 (< 0.001)
	Stakeholders	56.68 (20.36)	32.62 (19.35)	*F*(1, 163) = 59.78 (< 0.001)
5 (synergy)	Long-term	72.65 (17.12)	53.26 (25.63)	*F*(1, 87) = 17.69 (< 0.001)
	Stakeholders	69.34 (20.36)	50.26 (27.59)	*F*(1, 87) = 13.76 (< 0.001)
5 (no synergy)	Long-term	71.28 (17.21)	45.66 (20.56)	*F*(1, 96) = 43.99 (< 0.001)
	Stakeholders	72.51 (17.73)	34.14 (23.45)	*F*(1, 96) = 81.85 (< 0.001)

A one-way between subjects ANOVA (controlling for nationality in exp. 2) revealed that participants attributed more experience to Alpha when it was stakeholder-oriented (exp. 1: *M* = 4.94; *SD* = 1.37; exp. 2: *M* = 4.14; *SD* = 1.82) than when it was profit-oriented [exp. 1: *M* = 3.05; *SD* = 0.89; *F*(1, 81) = 55.23; *p* < 0.001; η^2^*_*p*_* = 0.408; exp. 2: *M* = 3.06; *SD* = 1.76; *F*(1, 88) = 7.84; *p* = 0.006; η^2^*_*p*_* = 0.084]. This supports hypothesis 1. Participants did not attribute more agency to Alpha when it was stakeholder-oriented (exp. 1: *M* = 4.80; *SD* = 1.28; exp. 2: *M* = 4.74; *SD* = 1.78) than when it was profit-oriented [exp. 1: *M* = 4.61; *SD* = 0.98; *F*(1, 81) = 0.61; *p* = 0.436; η^2^*_*p*_* = 0.008; exp. 2: *M* = 4.85; *SD* = 1.73; *F*(1, 88) = 0.07; *p* = .793; η^2^*_*p*_* = 0.001]. I ran a MANOVA for experiment 1 on each of the experience and agency items separately (see Table 2). The results were the same.

**TABLE 2:** Means, standard deviations, and significance levels of experience and agency items in experiment 1.

Mind dimension	Item	Stakeholder	Profit	

		*M* (*SD*)	*M* (*SD*)	*F* (*p*)
Experience	Emotion	4.68 (1.53)	2.71 (1.26)	*F*(1, 81) = 40.56 (< 0.001)
	Consciousness	5.20 (1.62)	3.38 (1.23)	*F*(1, 81) = 33.01 (< 0.001)
Agency	Intention	5.15 (1.46)	4.88 (1.21)	*F*(1, 81) = 0.83 (= 0.366)
	Free will	4.63 (1.37)	4.40 (1.71)	*F*(1, 81) = 0.41 (= 0.523)
	Mind of its own	4.65 (1.61)	4.55 (1.52)	*F*(1, 81) = 0.88 (= 0.768)

## Experiments 3 and 4

In experiments 1 and 2, I investigated the effect of a business orientation on experience and agency attributions to the firm. The results show that a firm’s orientation influences experience attributions, but not agency attributions. Experiments 3 and 4 aimed to investigate the effect of a firm’s orientation on moral standing attributions to the firm, mediated by experience attributions to the firm. Importantly, participants may also attribute experience or agency to the human stakeholders who are part of the firm (Ashforth et al., 2020; Quintelier et al., 2021). In order to explore if this influences moral standing attributions to the firm, I also included experience and agency attributions to employees. As described in the Procedures and Measures below, the vignettes were slightly adapted in order to avoid a linguistic association between the vignettes and moral standing items. The experience and agency measures were also improved to increase their reliability.

### Sample and Design

Based on previous experiments (Rai and Diermeier, 2015; Quintelier et al., 2021), I expected large effect sizes for the trade-off vignettes and large to moderate effect sizes for the no trade-off vignettes, which requires a sample size of 35–74 for a percentile bootstrap test for mediation (Fritz and MacKinnon, 2007). Participants were randomly assigned to a stakeholder- or profit-oriented vignette. For experiment 3, participants were recruited on www.clickworker.com and were compensated 0.7€, which is higher than in experiment 2 because this experiment contained additional items for experience, agency and moral standing. Sixty-one participants completed experiment 3 (36 men, *M*_*age*_ = 35.49; *SD* = 8.68; 28 stakeholder-oriented; Supplementary Data Sheet 3). After removing participants that gave a wrong answer to an attention check, 51 participants remained (31 men; *M*_*age*_ = 35.96; *SD* = 9.19; 22 stakeholder-oriented). For experiment 4, 174 participants, recruited on a university in the Netherlands, completed the experiment for course credit (131 men, *M*_*age*_ = 19.88; *SD* = 1.97; 86 stakeholder-oriented; Supplementary Data Sheet 4). After removing participants who gave a wrong answer to an attention check, 164 participants remained (122 men, *M*_*age*_ = 19.88; *SD* = 2.00; 81 stakeholder-oriented).

### Procedure and Measures

In experiments 1 and 2, the stakeholder-oriented vignettes and one attention check answer option contained the word “morally.” For experiments 3 and 4, the survey includes moral standing items, and two moral standing items also contain the word “morally.” In order to avoid a linguistic association between the stakeholder-oriented vignette and moral standing, I removed the word “morally” from the stakeholder-oriented vignette and the attention check answer option. The vignettes and attention checks were otherwise the same as in experiments 1 and 2.

In experiment 1, the reliability of organizational experience and agency was low. I therefore added all non-overlapping experience and agency items based on Tang and Gray (2018). The items for experience now were: “have feelings”; “have consciousness”; and “experience emotions.” The reliability of organizational experience was good (exp. 3: α = 0.916; exp. 4: α = 0.829). The items for agency now were: “have intentions”; “have free will”; “have a mind of its own”; “capable of planning”; and “capable of thinking.” The reliability for organizational agency was good to acceptable (exp. 3: α = 0.879; exp. 4: α = 0.770). To rule out the alternative explanation that participants interpreted these questions as experience and agency of the firm’s employees instead of the firm, I also included items measuring experience and agency attributions to Alpha’s employees. I used the same items as those measuring organizational experience and agency, except that I now asked to what extent Alpha’s employees had these characteristics. For employee experience, the reliability was excellent to good (exp. 3: α = 0.915; exp. 4: α = 0.863). For organizational agency reliability was excellent to good (exp 3: α = 0.922; exp. 4: α = 0.826).

For moral standing I adapted the five moral standing items from Piazza et al. (2014) to fit a corporate context. Participants rated the items on a seven-point Likert scale from “not at all” to “very much.” The items were: “How morally wrong do you think it would be for someone to harm Alpha”; “How morally wrong do you think it would be for someone to steal from Alpha”; “To what extent do you think Alpha deserves to be treated with compassion?”; “To what extent do you think Alpha deserves to be protected from harm?” and “If Alpha were in danger, how important would it be to protect Alpha?” The scale’s reliability was good (exp. 3: α = 0.887; exp. 4: α = 0.827). This was followed by realism and imaginability of the vignettes, the manipulation checks and demographic variables (year of birth, gender, nationality).

### Results

A MANOVA on the manipulation checks revealed that the manipulation worked (see Table 1). In experiment 3, the stakeholder-oriented vignette had a significantly higher proportion of German participants than the profit-oriented vignette (13 vs. 8; *p* = 0.011). I controlled for nationality in the analysis of experiment 3. There were no other significant differences in demographics or control questions between the stakeholder- and profit-oriented vignette.

**FIGURE 1:**
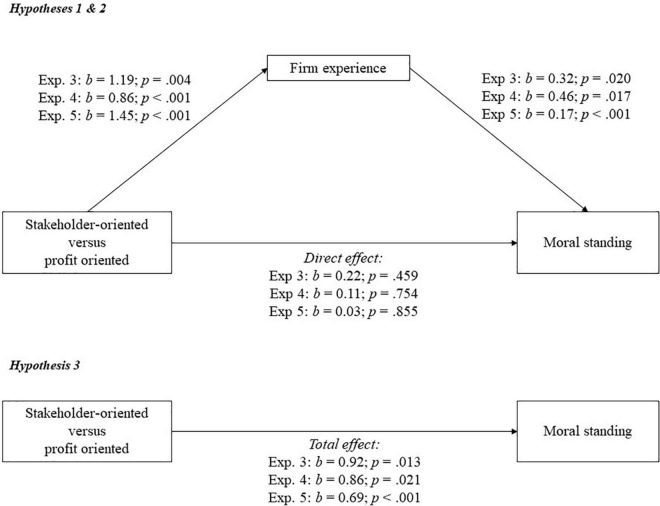
A model of mediation indicating that the relationship between business orientation and moral standing goes via experience attributions to the firm in experiments 3, 4, and 5.

I conducted a mediation analysis with firm experience and agency, and employee experience and agency, as mediators, and controlling for nationality in experiment 3 (model 4 of the PROCESS macro in SPSS, percentile bootstrap, 5,000 bootstrap samples, seed = 12,345, Hayes, 2013). The most important results for experiment 3–5 are summarized in Figure 1. Table 3 provides the means, standard deviations, and effects of orientation on all mediators and on the dependent variable, for all experiments. The analysis revealed that participants attributed more moral standing to the stakeholder-oriented firm (exp. 3: *M* = 5.62; *SD* = 1.08; exp. 4: *M* = 5.38; *SD* = 0.95) than to the profit-oriented firm (exp. 3: *M* = 4.82; *SD* = 1.26; *b* = 0.92; *p* = 0.013; exp. 4: *M* = 4.96; *SD* = 1.06; *b* = 0.86; *p* = 0.021; Figure 1). Participants attributed more experience to the stakeholder-oriented firm (exp. 3: *M* = 4.83; *SD* = 1.04; exp. 4: *M* = 4.81; *SD* = 1.15) than to the profit-oriented firm (exp. 3: *M* = 3.72; *SD* = 1.49; *b* = 1.19; *p* = 0.004; exp. 4: *M* = 3.96; *SD* = 1.18; *b* = 0.86; *p* < 0.001; Figure 1), and higher experience attributions to the firm increased moral standing attributions to the firm (exp. 3: *b* = 0.32; *p* = 0.020; exp. 4: *b* = 0.46; *p* = 0.017; Figure 1). The bootstrap analysis found that firm experience significantly mediated the effect of orientation on moral standing (exp. 3: *b* = 0.38; LLCI = 0.07; ULCI = 0.88; exp. 4: *b* = 0.39; LLCI = 0.013; ULCI = 0.86). There was no mediation via organizational agency (exp. 3: *b* = –0.11; LLCI = –0.39; ULCI = 0.06; exp. 4: *b* = 0.07; LLCI = –0.04; ULCI = 0.32) or employee agency (exp. 3: *b* = 0.34; LLCI = –0.04; ULCI = 0.91; exp. 4: *b* = 0.03; LLCI = –0.18; ULCI = 0.23). In experiment 4, but not in experiment 3, there was mediation via employee experience (exp. 3: *b* = 0.08; LLCI = –0.12; ULCI = 0.52; exp. 4: *b* = 0.26; LLCI = 0.008; ULCI = 0.65). There was no remaining direct effect (exp. 3: *b* = 0.22; *p* = 0.459; exp. 4: *b* = 0.11; *p* = 0.754; Figure 1), suggesting total mediation of the main effect. Rerunning the analysis with all participants, without control variables, or with different seed randomizers, gave similar results. These results support hypotheses 1, 2, and 3.

**TABLE 3:** Means, standard deviations, and significance levels of mediators and dependent variable in experiments 1–5.

Experiment	Variable	Stakeholder	Profit	

		*M* (*SD*)	*M* (*SD*)	*F* (*p*) or *b* (*p*)
1 (trade-off)	Firm experience	4.94 (1.37)	3.05 (0.89)	*F*(1, 81) = 55.23 (< 0.001)
	Firm agency	4.80 (1.28)	4.61 (0.98)	*F*(1, 81) = 0.61 (= 0.436)
2 (no trade-off)	Firm experience	4.14 (1.82)	3.06 (1.76)	*F*(1, 88) = 7.84 (= 0.006)
	Firm agency	4.74 (1.78)	4.85 (1.73)	*F*(1, 88) = 0.07 (= 0.793)
3 (trade-off)	Firm experience	4.83 (1.04)	3.72 (1.49)	*b* = 1.19 (= 0.004)
	Firm agency	5.26 (0.86)	4.88 (1.14)	*b* = 0.58 (= 0.058)
	Employee experience	5.35 (0.81)	4.99 (1.06)	*b* = 0.48 (= 0.103)
	Employee agency	5.22 (0.76)	4.63 (1.06)	*b* = 0.64 (= 0.027)
	Moral standing	5.62 (1.08)	4.82 (1.26)	*b* = 0.92 (= 0.013)
4 (no trade-off)	Firm experience	4.81 (1.15)	3.96 (1.18)	*b* = 0.86 (< 0.001)
	Firm agency	5.01 (0.86)	4.81 (1.03)	*b* = 0.19 (= 0.185)
	Employee experience	5.30 (1.05)	4.82 (1.13)	*b* = 0.48 (= 0.005)
	Employee agency	5.00 (0.86)	4.72 (1.09)	*b* = 0.28 (= 0.069)
	Moral standing	5.38 (0.95)	4.96 (1.06)	*b* = 0.86 (= 0.021)
5 (pooled)	Firm experience	5.08 (1.45)	3.62 (1.33)	*b* = 1.45 (< 0.001)
	Firm agency	5.25 (1.19)	4.87 (1.08)	*b* = 0.38 (= 0.023)
	Employee experience	5.57 (1.27)	4.75 (1.20)	*b* = 0.82 (< 0.001)
	Employee agency	5.35 (1.12)	4.71 (1.15)	*b* = 0.64 (< 0.001)
	Moral standing	5.38 (1.19)	4.68 (1.16)	*b* = 0.69 (< 0.001)

## Experiment 5

Experiments 1–4 made use of plain text, theory-driven descriptions of a grocery store. However, in real life, individuals are likely to observe firms via specific media such as their website, containing self-presentations that often highlight financial and stakeholder value created in synergy. In order to increase the ecological validity, participants were now presented with a mock website. In order to expand the results to stakeholder- and profit-oriented firms featuring synergy, the mock website focused either on (1) stakeholder elements, in synergy with profit elements, (2) profit elements, in synergy with stakeholder elements, (3) stakeholder elements only, or (4) profit elements only. All data were collected in one effort, allowing for comparison between all conditions.

### Sample and Design

Two-hundred and two participants, recruited on clickworker and compensated 0.7€, completed the experiment (98 men, *M*_*age*_ = 38.04; *SD* = 10.81; 101 stakeholder-oriented; 98 synergy; Supplementary Data Sheet 5). Participants were randomly assigned to a stakeholder- or profit-oriented vignette, which either featured synergy or no synergy; thus using a 2 × 2 between-subjects factorial design. After removing participants who gave a wrong answer to an attention check, 185 participants remained (87 men; *M*_*age*_ = 37.91; *SD* = 10.82; 93 stakeholder-oriented; 88 synergy).

### Procedure and Measures

For describing the stakeholder orientation, I selected and adapted stakeholder elements found in the communications of Patagonia and Southwest Airlines, and for the profit orientation I selected and adapted profit elements found in the communications of Inditex and Ryanair. Southwest Airlines and Ryanair have been used to exemplify stakeholder vs. profit elements before (Bridoux and Stoelhorst, 2014). Patagonia, as a benefit corporation, has a “duty to consider and pursue the interests of a variety of stakeholders” (Mcdonnell, 2014, p. 22), making it a real-life example of a firm featuring stakeholder elements (Murray, 2012). Inditex is well-known for its short-term production (Zott and Amit, 2010) and arms-length transactions (Sancha et al., 2016), making it a real-life example of a firm featuring profit elements. Several vignettes were pre-tested on realism and imaginability. The text of the final vignette, depicted on a mock website, is described in Table 4.

**TABLE 4:** Vignette texts for experiment 5 for the stakeholder-oriented, profit-oriented, synergy, and no-synergy vignettes.

Introduction	
Meet Loco, your local supermarket What do we stand for?	

**Stakeholder (synergy)**	**Profit (synergy)**

Loco has a heart for food. We aim to benefit all our stakeholders. (We believe that profit happens after we benefit our stakeholders.)	Loco strives for excellence. We aim to be the market leader. (We believe that our stakeholders benefit when we maximize profit.)

What are we doing?	

We invest in strong relationships with our suppliers. (This strategy translates into long-term growth.)	We deliver double-digit quarterly growth. (To realize this, we rely on effective relationships with our suppliers.)
We treat our employees with respect, for instance by protecting their work-life balance. (This respect leads to sustainable financial success.)	We achieve above average financial success. (This success supports the respectful treatment of our employees, for instance by protecting their work-life balance.)
At Loco we continually offer free healthy food advice for our customers. (This advice led to an increase in the sales of our products.)	At Loco we aim for a 36% increase in the sales of our products. (To attain this goal we temporarily offer free healthy food advice for our customers.)
Loco sources fresh food from local community gardens. (We organize events to support their coordination.)	Loco has cut packaging costs by 18%. We did this by sourcing fresh food from local community gardens.

The vignette was followed by two factual attention checks (Kane and Barabas, 2019), where participants had to select the phrase that was part of the vignette they had just read. For the first question, the options were: “we aim to benefit all our stakeholders” (correct for stakeholder-oriented) and “we aim to be the market leader” (correct for profit-oriented). For the second question, the options were: “we deliver double-digit quarterly growth” (correct for profit-oriented) and “we invest in strong relationships with our suppliers” (correct for stakeholder-oriented). This was followed by the items for organizational experience and agency, employee experience and agency, and moral standing, which were the same as before. The reliability of the measures was good (firm experience: α = 0.885; firm agency: α = 0.844; employee experience: α = 0.878; employee agency: α = 0.889; moral standing: α = 0.830). This was followed by realism and imaginability of the vignettes, the manipulation checks and demographic variables (year of birth, gender, nationality).

### Results

A MANOVA on the manipulation checks revealed that the manipulation worked (see Table 1): the stakeholder-oriented vignette was perceived as more stakeholder- and long-term-oriented than the profit-oriented vignette, both in the synergy and no-synergy condition. There were no significant differences in realism, imaginability, or demographics between the stakeholder- and profit-oriented vignettes, both in the synergy or no-synergy condition.

Exploring the data (model 7 of the PROCESS macro in SPSS, percentile bootstrap, 5,000 bootstrap samples, seed = 12,345, Hayes, 2013) showed that experience attributions to the firm mediated the effect of business orientation on moral standing attributions in both the synergy and no-synergy condition. I therefore pooled the synergy and no-synergy conditions and conducted a mediation analysis with firm experience and agency, and employee experience and agency, as mediators, on all the data (model 4 of the PROCESS macro in SPSS, percentile bootstrap, 5,000 bootstrap samples, seed = 12,345, Hayes, 2013). This analysis revealed that participants attributed more moral standing to the stakeholder-oriented firm (*M* = 5.38; *SD* = 1.19) than to the profit-oriented firm (*M* = 4.68; *SD* = 1.16; *b* = 0.69; *p* < 0.001; Figure 1). Participants attributed more experience to the stakeholder-oriented firm (*M* = 5.08; *SD* = 1.45) than to the profit-oriented firm (*M* = 3.62; *SD* = 1.33; *b* = 1.45; *p* < 0.001; Figure 1), and higher experience attributions to the firm increased moral standing attributions (*b* = 0.17; *p* < 0.001; Figure 1). The bootstrap analysis found that firm experience significantly mediated the effect of orientation on moral standing (*b* = 0.37; LLCI = 0.08; ULCI = 0.65). There was no mediation via employee agency (*b* = –0.02; LLCI = –0.16; ULCI = 0.09). There was mediation via firm agency (*b* = 0.16; LLCI = 0.03; ULCI = 0.33), and employee experience (*b* = 0.16; LLCI = 0.02; ULCI = 0.33). There was no remaining direct effect (*b* = 0.03; *p* = 0.855; Figure 1), suggesting total mediation of the main effect. Rerunning the analysis with all participants, with and without control variables, or with different seed randomizers, gave similar results. These results support hypotheses 1, 2, and 3.

## Discussion

Moral standing attributions to firms are prevalent, consequential, and often the subject of opposition and controversy, as is illustrated in the legal, philosophical, and public debate about corporate moral standing (Garrett, 2014; Iuliano, 2015; Levitt, 2015; Silver. 2019). However, there is little empirical research about the psychological factors influencing moral standing attributions to corporations (for an exception, see Mentovich et al., 2016). In this paper, five experiments tested, and found, that experience attributions to firms are important to understand moral standing attributions to firms, and that observing a firm as stakeholder-oriented increases experience attributions to the firm. These effects replicated when a business orientation was operationalized with or without a trade-off or synergy. While some experiments also found an effect via firm agency or employee experience, these effects were not consistent, and were in all cases smaller than the effect via firm experience. The relevance of experience attributions for moral standing attributions to firms has implications for business ethics and stakeholder theory.

### Business Ethics and Corporate Moral Standing

Business ethicists (as well as legal scholars) ask normative questions about why we *should* grant moral standing to corporations. This paper follows an empirical approach, not a normative approach. The question here is why individuals *do* grant moral standing to firms. Importantly, empirical descriptions of the world cannot be used to deductively infer normative conclusions (Hume, 1739–1740; Moore, 1903). Nonetheless, empirical findings about what people do can have normative implications, or implications about what people should do, in other, non-deductive ways (Nesteruk, 1992; Schurz, 1997; Harris and Freeman, 2008; Quintelier et al., 2011; Quintelier and Zijlstra, 2014; Werhane, 2019; Prochownik, 2021). Specifically, the empirical findings in this paper suggest that corporate experience is an important, but relatively overlooked, argument in the normative debate about corporate moral standing.

In the normative debate about corporate moral standing, stakeholders’ experience and agency as well as corporate agency, feature as important reasons to grant moral standing to corporations (Silver, 2019). For example, Blair and Pollman (2014) argue that corporations usually have been granted legal rights because they are seen as an “association of persons acting together” (Blair and Pollman, 2014; Blair, 2015, p. 421), suggesting that stakeholders’ agency influences corporate moral standing. Turning to corporate agency, Sepinwall (2015) argues that if corporations are moral *agents*, then they are moral persons bearing moral rights. However, this latter argument is contradicted by Silver (2019, pp. 253–254), who reviews the state of the business ethics debate and notes that “[m]any authors are ready to ascribe agential capacities to corporations, but they resist seeing corporations as potentially deserving certain rights.” This yields an interesting puzzle: why do some authors see corporate agency as an argument in favor of firm moral standing, while other authors do not see a link between corporate agency and moral standing?

The theory and findings in this paper point to the possibility that experience attributions are part of the explanation. The theory of dyadic morality suggests that experience attributions are essential for triggering moral standing attributions (Gray et al., 2012). Previous findings (Sytsma and Machery, 2012), as well as the findings in this paper, find that experience attributions consistently influence moral standing attributions, while agency attributions only sometimes have an effect. It is therefore possible that, first, experience attributions are a necessary condition for eliciting moral standing attributions, and second, only when experience attributions are high enough do agency attributions influence moral standing attributions. This possibility is in line with the reported findings. In experiment 5, experience attributions to the stakeholder-oriented firm were higher than in every other condition of all the experiments (see Table 3). Experiment 5 is also the only experiment where firm agency influences moral standing. To further explore this possibility, I conducted a moderated mediation model with experience attributions to the firm as moderator (model 7 of the PROCESS macro in SPSS, percentile bootstrap, Hayes, 2013). I found that in experiments 3, 4, and 5, agency attributions indeed have a more positive (or less negative) effect on moral standing attributions if experience attributions are higher. While these effects are not significant, they do suggest that experience attributions are essential for moral standing attributions while agency attributions only contribute to moral standing attributions if experience attributions are high enough. In other words, the suggestion is that low experience attributions to an entity are sufficient to deny moral standing to that entity; if experience attributions are low, agency attributions do not influence moral standing attributions. Future research can investigate this possibility.

This possible explanation sheds new light on the normative debate. While some legal scholars defend that corporations have emotions and deserve protection (e.g., Iuliano, 2015), business ethicists tend to deny that corporations have emotions, and denying corporate experience seems sufficient to deny corporate moral standing. This is in line with Silver (2019, pp. 253–254) interpretation of the literature when he writes that “few proponents of corporate agency even consider the possibility that corporations might be moral patients” (patiency being a concept closely related to experience). This is coupled to intuitions about moral standing that “the idea that a corporation like McDonalds has moral rights deserving protection sounds absurd” (Silver, 2019, p. 253). Likewise, Hess dismisses that corporations are deserving of rights and protections, because they do not have experiences that make them vulnerable; in her view, corporations having agency is not sufficient to grant them moral standing (Hess, 2013, pp. 333–334). Likewise, the illustrations in the beginning of this paper featured normative arguments for or against corporate moral standing that hinged on corporate experience only. The relation between experience and moral standing seems uncontroversial, and perhaps therefore in little need of philosophical elaboration.

In contrast, the relation between agency and moral standing is more fickle, but also more extensively discussed. Silver (2019, pp. 253–254) reviews the state of the business ethics debate and notes that agency tends to be an argument in favor of moral standing, but at the same time, “[m]any authors are ready to ascribe agential capacities to corporations, but they resist seeing corporations as potentially deserving certain rights.” As a consequence, in the normative debate, various arguments are brought to bear on why agency does or does not matter for moral standing. However, it is relatively underexplored to what extent assumptions about corporate experience underly this disagreement. It is possible that, also in the normative debate, an entity’s level of agency only matters when it has a minimum level of experience. I therefore suggest that business ethicists, when discussing corporate moral standing, explicate their assumptions about corporate experience, in order to unearth potentially important assumptions.

### Stakeholder Theory, Experience, and Moral Standing Attributions to Firms

The findings in this paper can shed new light on previous empirical findings, and suggest that stakeholder theory can expand its scope by integrating insights from the psychology of humanization. Rai and Diermeier (2015) argue that individuals tend not to attribute experience to (for-profit) companies. It is only when exploring a broader range of organizations, that they find that experience attributions do vary depending on the type of organization. Non-profits rank higher than small businesses, which rank higher than multinational companies in experience attributions. While interesting, they did not investigate which factors caused this variation. Likewise, Mentovich et al. (2016) find that specific rights attributions to organizations vary, with family owned companies and mental health centers ranking higher than local chains, which rank higher than national chains in rights attributions. They suggest on the basis of their findings that individuals attribute more rights to smaller organizations and to less profit-driven organizations. The findings in this paper offer a first explanation of these findings. They show, first, that certain kinds of firms do reliably elicit higher experience and moral standing attributions; and second, that the balance between stakeholder and profit elements influences experience and moral standing attributions. This is a new insight in stakeholder theory.

The findings in this paper also raise novel questions for stakeholder scholars. Stakeholder scholars argue that stakeholder-oriented firms are positively related to moral consideration for the interests of *stakeholders* (Wicks et al., 1994; Phillips and Reichart, 2000; McVea and Freeman, 2005; Harris and Freeman, 2008; Freeman et al., 2010; Quintelier et al., 2021). So far, stakeholder scholars did not investigate the relation between a stakeholder orientation and moral standing of the *firm* instead of its stakeholders. The findings in this paper lead to the conclusion that stakeholder-oriented firms are positively related to moral consideration for stakeholders’ interests as well as to moral standing attributions to firms. This raises the possibility that individuals observing a stakeholder-oriented firm will show more moral consideration for stakeholders (Quintelier et al., 2021), while at the same time attributing more moral standing to the firm. This begs the question how this will play out in situations where the interests of the firm and the interests of its stakeholders are in conflict with each other.

At first sight, the current state of the findings suggests that such firm-stakeholder conflicts in stakeholder-oriented firms will elicit stronger moral concerns in favor of both sides of the conflict, leading simply to stronger dilemmas. For instance, Antoni et al. (2020) find that dilemmas about caring for work (i.e., the firm) vs. co-workers (the firm’s stakeholders) play out differently in a multinational vs. a child protection service. In the multinational, care dilemmas are suppressed, while in the child protection service, care dilemmas are a constant struggle. To the extent that a child protection service is more stakeholder-oriented than a multinational, this supports that stakeholder-oriented firms will elicit stronger moral dilemmas.

However, a closer reading reveals that these conflicts might benefit stakeholders. First of all, the work of Antoni et al. (2020) also shows that employees in the multinational sacrifice employees and prioritize work. In contrast, employees in the child protection service seem to integrate both interests. In addition, Mentovich et al. (2016) suggests that moral standing attributions to firms are driven by perceptions that the firm is supporting the interests of its stakeholders. Therefore, in stakeholder-oriented firms, firm-stakeholder conflicts would lead to stronger moral dilemmas that are eventually decided in favor of stakeholders.

This also leads to another interpretation of the current findings. It is possible that individuals attribute more moral standing to stakeholder-oriented firms, not because they want to protect the firm in a conflict with its stakeholders, but because they want to protect the stakeholders against the negative outcomes if something bad happens to the firm. This is also in line with some arguments in business ethics, that legal rights for corporations are instrumental to corporations’ actions *to protect the rights of their stakeholders* (e.g., Bishop, 2012; Pasternak, 2017). Likewise, Goodstein and Wicks (2007) develop a normative argument that stakeholders should consider the interests of firms, at least “to the extent that firms are responsible for fulfilling duties to stakeholders” (Goodstein and Wicks, 2007, p. 376; see also Phillips, 1997). Hence, while the current paper finds a positive relation between a stakeholder orientation and moral standing attributions to the firm, it is possible that a concern for stakeholders is part of the explanation. Future work can investigate this possibility.

### Strengths, Limitations, and Future Research

This paper investigated the effect of a business orientation on experience and moral standing attributions to firms. The scope of the experiments was limited to specific theoretical predictions about causal relationships at the individual level. Experiments are well-suited to test theory, causal relationships, and individual-level mechanisms. Within this scope, the set of experiments in this paper provide evidence for the theoretically predicted, causal link, between a business orientation and individuals’ experience and moral standing attributions. In addition, the last experiment replicated the results while increasing the ecological validity of the design. These results provide a strong basis to expand the scope of future studies. Specifically, while the present study was limited to immediate effects in the context of firms, future work can investigate the long-term effects of a business orientation, or the effects of a business orientation in other types of organizations. For instance, surveys could investigate how individuals’ experience and agency attributions change after they start a contractual relationship with a stakeholder- vs. a profit-oriented firm. It is also possible to modify a stakeholder and profit orientation for small and large businesses, or for non-profits, in order to generalize the results and investigate potentially moderating effects of firm size and the kind of corporation.

Another limitation related to the scope of this study is that I focus only on experience and moral standing attributions. I did not develop predictions about agency attributions and moral responsibility. However, studies find that individuals’ agency attributions to an entity are related to individuals’ moral responsibility attributions to that entity (Gray et al., 2007). Likewise, as Ruiz-Palomino et al. (2011) note, business ethicists have argued that corporations do not have moral responsibility due to their lack of agency. Investigating the relationship between agency attributions and moral responsibility attributions to firms, possibly in the context of stakeholder- vs. profit-oriented firms, would paint a more complete picture about individuals’ moral cognition in a business context.

This study investigated moral standing attributions to firms, but other abstract entities are deserving of moral standing as well. While most stakeholder scholars limit discussions about moral consideration, rights, and protections, to human stakeholders (Phillips and Reichart, 2000), others, such as Starik (1995) and Driscoll and Starik (2004), argue that the natural environment is also deserving of moral consideration. This is not a hypothetical possibility, as some corporations do take the interests of the environment into account (Stubbs and Cocklin, 2008). Given the state of the natural environment (Rockström et al., 2009; Whiteman et al., 2013), it is also important to elicit protective attitudes toward the environment. Increasing experience attributions to the natural environment might be a pragmatic way to increase moral standing attributions to the environment. The current finding that abstract entities such as firms can elicit experience and moral standing attributions, bring hope that this might also be achieved for the environment.

## Conclusion

In this paper I argued that individuals attribute more moral standing to stakeholder-oriented than to profit-oriented firms, because individuals attribute more experience (such as feelings) to stakeholder-oriented firms. The psychological processes unearthed in this paper provide a better understanding of the philosophical, legal and public debate about corporate moral standing. The findings also shed new light on previous empirical findings, and bring the debate about corporate moral standing to the heart of stakeholder theory. In the future, business ethics and stakeholder theory can benefit by integrating more insights from the psychology of humanization. Future research can also expand this work by investigating moral standing attributions to other abstract entities in need of protection, such as the natural environment.

## Data Availability Statement

The original contributions presented in the study are included in the article/Supplementary Material, further inquiries can be directed to the corresponding author/s.

## Ethics Statement

The studies involving human participants were reviewed and approved by the Research Ethical Review Board Vrije Universiteit Amsterdam. The patients/participants provided their written informed consent to participate in this study.

## Author Contributions

KQ: conceptualization, methodology, validation, formal analysis, investigation, resources, data curation, writing, and project administration.

## Conflict of Interest

The author declares that the research was conducted in the absence of any commercial or financial relationships that could be construed as a potential conflict of interest.

## Publisher’s Note

All claims expressed in this article are solely those of the authors and do not necessarily represent those of their affiliated organizations, or those of the publisher, the editors and the reviewers. Any product that may be evaluated in this article, or claim that may be made by its manufacturer, is not guaranteed or endorsed by the publisher.
